# Examining the involvement of guardians of children with acute lymphoblastic leukemia in Tanzania as public contributors to inform the design and conduct of the GuardiansCan project: A mixed‐methods study protocol

**DOI:** 10.1002/cam4.70034

**Published:** 2024-07-23

**Authors:** Faraja Chiwanga, Joanne Woodford, Golden Masika, David A. Richards, Victor Savi, Louise von Essen

**Affiliations:** ^1^ Muhimbili National Hospital, Research and Consultancy Unit Dar es Salaam United Republic of Tanzania; ^2^ Department of Women's and Children's Health, Healthcare Sciences and e‐Health Uppsala University Uppsala Sweden; ^3^ Department of Clinical Nursing University of Dodoma Dodoma United Republic of Tanzania; ^4^ Department of Health and Caring Sciences Western Norway University of Applied Sciences Bergen Norway

**Keywords:** childhood cancer, LMICs, mHealth, public contribution

## Abstract

**Background:**

Public contribution in research can lead to the design and conduct of more feasible and relevant research. However, our understanding of the acceptability and feasibility of public contribution and the evidence base regarding its impact in low‐ and middle‐income countries (LMICs) is limited.

**Methods:**

In this study protocol, we describe a mixed‐method examination of public contribution activities in the GuardiansCan project. The GuardiansCan project aims to respond to Tanzanian guardians' poor adherence to children's follow‐up care after treatment for acute lymphoblastic leukemia (ALL) with the help of Mobile Health technology. We aim to: (1) involve guardians of children treated for ALL as Guardians Advisory Board (GAB) members in the managing and undertaking, analysis and interpretation, and dissemination phases of the GuardiansCan project; and (2) examine the acceptability, feasibility, and perceived impact of GAB members' contribution to the GuardiansCan project from the perspective of the GAB members and public contribution coordinators. We will recruit six to eight guardians of children treated for ALL to the GAB. We will hold workshops where GAB members contribute to all project phases. Using impact logs, we will record GAB workshop activities and the perceived impact of these activities. We will interview GAB members and public contribution coordinators 6 months after establishing the GAB, and at the end of each study within the project, to examine the acceptability, feasibility, and perceived impact of public contribution activities.

**Discussion:**

We expect GAB contribution to increase project quality and relevance, and inform how to best embed public contribution in research in LMICs.

## 1 INTRODUCTION

Cancer is a major global cause of death during childhood.^1^, ^2^ In many low‐ and middle‐income countries (LMICs), survival rates are around 20%,^3^ compared to over 80% in high‐income countries (HICs).^4^ If treatment inequalities remain unaddressed, 84.1% of the 11.1 million children projected not to survive childhood cancer between 2020 and 2050 will be from LMICs.^5^ Avoidable deaths result from factors such as lack of diagnosis, misdiagnosis, delayed diagnosis, difficulties accessing care, use of traditional and complementary medicine, financial hardship, lack of survivorship care, and treatment abandonment.^5^, ^6^, ^7^

A leading factor associated with poor childhood cancer survival rates in LMICs is treatment abandonment.^8^, ^9^ Our research in Tanzania shows guardians of children with cancer report unmet needs related to health provider communication, information about childhood cancer, and emotional support that may impact adherence to treatment and follow‐up care.^10^ Providing follow‐up care reminders, information, and emotional support via a mobile health (mHealth) intervention may be a promising strategy for improving guardians' adherence to treatment and follow‐up care,^11^, ^12^ and ultimately childhood cancer survival rates. Therefore, we are currently undertaking the GuardiansCan project, aiming to respond to Tanzanian guardians' poor adherence to children's follow‐up care after treatment for acute lymphoblastic leukemia (ALL) with the help of mHealth technology.^13^ ALL is targeted due to potential challenges adhering to treatment and follow‐up care during the long‐term maintenance period (2–3 years), given treatment length and complexity are associated with nonadherence.^14^

Public contribution in research is becoming increasingly recognized and prioritized when designing and undertaking healthcare research. Therefore, as a first step, we will establish a Guardians Advisory Board (GAB) to implement public contribution.^15^, ^16^, ^17^ Public contribution in research is commonly defined as research “carried out “*with*” or “*by*” members of the public rather than “*to*,” “*about*,” or “*for*” them.”^18^ Members of the public (e.g., the general public, current or former patients, and informal caregivers/family members) are active research partners, contributing to decisions concerning how research is prioritized, designed, conducted, analyzed, interpreted, and disseminated.^19^ Within several HICs, public contribution in research is routinely adopted by funding bodies, researchers, and policymakers^20^, ^21^ and is becoming increasingly common in cancer research.^22^

Routine adoption of public contribution in research is associated with the growing evidence‐based concerning its impact.^23^, ^24^ Identified impacts include setting important and relevant research questions, improving recruitment rates, developing understandable participant‐facing study materials,^25^, ^26^, ^27^ improving the rigor and validity of data analysis and interpretation,^28^ and developing wider dissemination and communication strategies.^29^ Public contribution in research also improves knowledge among, and empowers, public contributors.^25^

Identified impacts in HCs may be amplified when used in settings less used to these approaches. Research in LMICs has been heavily influenced by the Neocolonialist model,^30^ whereby Western epistemologies have dominated research capacity building.^31^ However, this focus may fail to generate contextualized knowledge.^32^ Neglecting to contextualize research, for example, ignoring sociocultural and economic factors and local needs and preferences, may hinder research success in LMICs. Embedding public contribution in research represents one way to generate contextualized knowledge and facilitate conducting more appropriate and meaningful research in LMICs.^33^, ^34^ However, public contribution in research in LMICs remains in its infancy.^34^, ^35^, ^36^, ^37^ Researchers need to better understand how to implement public contribution and examine the acceptability, feasibility, and impact of public contribution in this setting.

### 1.1 Aims

The current paper describes the protocol for Study I of the GuardiansCan project. We aim to: (1) involve guardians of children treated for ALL as GAB members in the managing and undertaking, analysis and interpretation, and dissemination phases of the GuardiansCan project and (2) examine the acceptability, feasibility, and perceived impact of GAB members' contribution to the GuardiansCan project from the perspective of the GAB members and public contribution coordinators (hereafter referred to as coordinators).

## 2 METHODS

### 2.1 Overview

**FIGURE 1:**
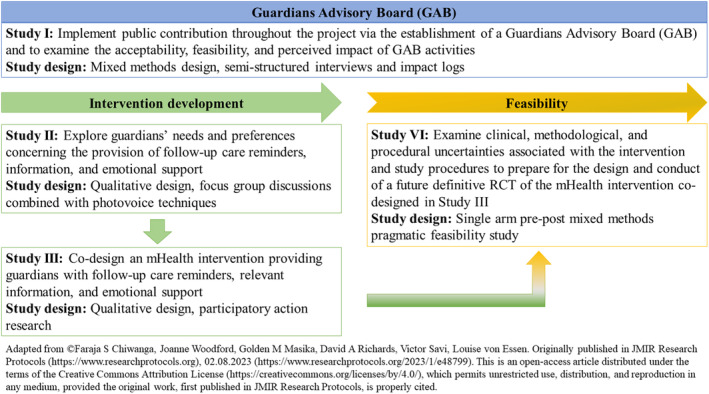
Overview of the GuardiansCan project.

Following the Medical Research Council (MRC) Framework for developing and evaluating complex interventions,^38^ we will undertake the GuardiansCan project to develop and test an mHealth intervention. Our overall goal is to increase guardians' adherence to children's medications and follow‐up visits and, second, to decrease their psychological distress. Overall objectives for Study I, II, III, and IV of the project can be seen in Figure 1 and a detailed overview has been published.^13^

We have developed the protocol for Study I following the Guidance for Reporting Involvement of Patients and the Public checklist (GRIPP2; see online supplementary Appendix S1)^39^ and informed by our research on involving parents of children treated for cancer in Sweden as public contributors.^40^

### 2.2 Public involvement framework

We will adopt a study‐focused framework for involving GAB members in the project^20^ with public contribution activities including: (1) managing and undertaking the project, such as designing Study II, III, and IV procedures, for example, developing recruitment and retention strategies, codesigning study material (i.e., information sheets and consent forms), and identifying acceptable and feasible data collection procedures;^41^, ^42^ (2) analyzing and interpreting results from Study II, III, and IV;^28^, ^43^, ^44^ and (3) disseminating results (e.g., codesigning plain language research summaries, developing local dissemination strategies including public exhibitions^29^, ^45^ and distributing results within GAB members informal networks).^46^

### 2.3 Setting

We will coordinate public contribution activities from Muhimbili National Hospital (MNH) in Dar es Salaam, Tanzania. Initially, public contribution activities will be held at MNH. However, the location may change as informed by GAB members' preferences, for example, if GAB members prefer to meet outside of a hospital setting. Following recommendations for conducting public contribution activities in LMIC settings,^34^ Tanzania research team members will coordinate public contribution activities. Coordinators have received training on public contribution from Swedish research team members during two face‐to‐face workshops in Tanzania and will receive ongoing supervision.

### 2.4 GAB members

To be eligible, GAB members must be guardians of a child discharged within the last 3–36 months from MNH after completing the main treatment for ALL (i.e., induction treatment phase), ≥18 years old, speak Kiswahili, have completed primary school education International Standard Classification of Education ([ISCED] level 1) to postsecondary nontertiary education ([ISCED] level 4), and currently reside in one of seven regions in Tanzania (Dar es Salaam, Dodoma, Iringa, Lindi, Morogoro, Mtwara, and Pwani) or the island of Zanzibar. Geographical locations were selected to facilitate the contribution of guardians from urban and rural areas with different cultural backgrounds represented within Tanzania, and accessibility (i.e., possible to travel via public transport) to Dar es Salaam.

Guardians unable to communicate in Kiswahili will be excluded. Whilst Tanzania is a multilingual country with over 125 languages, Kiswahili is the national language^47^ and due to resource limitations providing interpretation/translation services for other spoken languages is not possible. Guardians with a tertiary education will be excluded, given low education level is associated with treatment abandonment^48^ and guardians with low education levels may have different intervention needs. Furthermore, a wider criticism of public contribution is that contributors are typically highly educated and do not represent more diverse populations who may have different experiences, perspectives, and needs.^49^

### 2.5 Recruitment of GAB members

We will use a purposive sampling approach. Recruitment will be organized by coordinators in collaboration with pediatric oncologists/pediatric oncology nurses at the MNH Pediatric Oncology Unit. Oncologists/nurses will identify potential GAB members via MNH patient records. A coordinator will call identified guardians to explore inclusion criteria, explain the purpose of the GAB, and inform about the reimbursement plan (i.e., travel and living costs and an allowance equivalent to the government daily subsistence allowance for ISCED level 4 personnel). Initial interest, ability to travel for workshops and potentially stay overnight in Dar es Salaam, barriers to participation, and whether barriers might be overcome will also be explored. Coordinators will inform guardians that around 12 interested guardians will be invited to a recruitment meeting at MNH (≈2 h). Coordinators will also inform guardians that the GAB will include six to eight members with variation in gender, relationship to the child with ALL (e.g., parents, grandparents), educational level, and location lived in, and therefore not all guardians invited to the recruitment meeting will be invited into the GAB.

In the recruitment meeting, coordinators will explain the purpose of the GAB, the planned structure and frequency of GAB workshops, and explore guardians' expectations and motivations for contribution. After the meeting, guardians will be invited to lunch and thereafter to an individual meeting with the coordinators. Before individual meetings, coordinators and LvE (face‐to‐face or via Zoom) will select six to eight guardians to be invited to the GAB. Decisions and reasons for being invited or not invited to the GAB will be provided by coordinators during the individual meetings. Coordinators will provide those invited with written information and a consent form. After signing the consent form, GAB members will complete a background questionnaire (e.g., gender, age, relationship status, relationship to the child with cancer, educational background, and location lived in).

Given the GAB will be involved throughout the GuardiansCan project, withdrawal by some members is anticipated. We will replace GAB members who withdraw following the aforementioned recruitment procedure or via individual recruitment meetings.

### 2.6 GAB structure and workshops

Guardians Advisory Board workshops will be arranged and facilitated by coordinators. Coordinators will maintain regular contact with GAB members via telephone, e‐mail, video call, or smartphone messenger service, and share necessary workshop materials.

As suggested by the Tanzanian research team members, we will hold face‐to‐face GAB workshops at MNH to facilitate communication and the use of creative public contribution methods,^50^ such as drawings, storyboards, and/or craft activities.^49^ However, location may change based on GAB members' preferences. Each workshop is anticipated to last approximately 4 hours. Lunch and refreshments will be provided. We will plan regular breaks and provide additional breaks (i.e., prayer time for Muslim GAB members) if required. Societal macrostructures (e.g., patriarchy) may also inform changes for example, we will organize same‐gender events if necessary to overcome gender‐related concerns or where guardians voice a preference.^49^

We will hold a minimum of four workshops to inform the design and conduct of Study II (Figure 1), with ad‐hoc workshops or individual meetings held for additional contribution activities that arise. We anticipate holding a minimum of four face‐to‐face workshops to inform the design and conduct of Study III and IV, respectively (Figure 1).

Where necessary, we will provide alternative communication options (e.g., telephone, e‐mail, video‐call, or smartphone messenger service), for example, if a GAB member cannot attend a scheduled workshop. If GAB members fail to attend workshops and/or meetings, a coordinator will attempt to contact them to explore their reasons for not attending, as well as their ability, motivation, and willingness to be a public contributor, and remind them of their right to withdraw.

Preliminary workshop topics suggested to inform the design and conduct of Study II are outlined in Table 1. Due to the iterative nature of the GuardiansCan project, workshop topics are not yet outlined for Study III and IV. We will be open to adapting public contribution activities to the local context in collaboration with GAB members.^34^

**TABLE 1:** Preliminary Guardians Advisory Board (GAB) workshop topics to inform the design and conduct of Study II as planned for now.

Workshop	Main topic	Preliminary subtopics
1	Introduction to the GuardiansCan project and the GAB	Introduction to the GAB Introduction to the GuardiansCan project Introduction to public contribution in research GAB members' expectations GAB structure and location Communication plan for the project and forthcoming results Other topics
2	Understanding and initial planning for Study II	Recruitment of study participants Introduction to using photovoice techniques and to focus group discussions Other topics
3	Further planning for Study II	Written information and consent forms Frequency and setting of focus group discussions Topic guides for focus group discussions Other topics
4	Final planning for Study II	Ethical considerations, such as privacy, identity protection, and use of sensitive information Using cameras and Photovoice techniques Other topics

Abbreviation: GAB, Guardians Advisory Board.

### 2.7 Data collection

#### 2.7.1 Impact logs

During and after each workshop, coordinators will record public contribution activities and perceived impacts (i.e., changes to the research and potential benefits and harms) in an impact log.^40^, ^51^ Impact logs include the date, who was involved (i.e., those present, missing, and apologies), discussion (i.e., suggestions and recommendations from GAB members), impacts on research (i.e., potential impacts on the project), impacts on guardians (i.e., potential impacts on GAB members), impacts on coordinators (i.e., potential impacts on contributors), and other comments. Coordinators will present the impact log from the previous workshop at the beginning of the next workshop to gain GAB members' feedback on content and ensure accuracy. Workshop discussions will be audio‐recorded to ensure the accuracy of impact log content but will not be transcribed.

#### 2.7.2 Semistructured interviews

Research team members fluent in Kiswahili but not involved in GAB workshops will interview GAB members and coordinators (face‐to‐face or via the telephone) to explore the acceptability, feasibility, and perceived impact of GAB activities. Interview guides, informed by previous research,^40^, ^51^ will explore reasons for becoming a GAB member, public contribution activities carried out, the purpose of the GAB, importance and impact of the GAB, barriers and facilitators working with the GAB, benefits and harms from working with the GAB, and suggestions for improvements.

We will conduct interviews 6 months after the GAB has been formed and at the end of Study II, III, and IV. We will invite all GAB members and coordinators to participate. We will audio‐record interviews and transcribe recordings verbatim. Kiswahili transcripts will be translated to English and subsequently back‐translated by an external translation company.

### 2.8 Data analysis

We will adopt a convergent parallel design with quantitative data (impact logs) and qualitative data (impact logs and semistructured interviews) collected concurrently, analyzed separately, and integrated at the point of interpretation where areas of convergence/divergence will be discussed.

#### 2.8.1 Impact logs

Two research team members not involved in GAB workshops will read, extract, and summarize impacts recorded in impact logs. Suggestions and recommendations from GAB members will be categorized (e.g., recruitment strategies, data collection, dissemination) and counted. We will calculate the percentage of suggestions and recommendations subsequently implemented for each category.^40^ We will present summaries of impacts to GAB members and coordinators to make sense of and interpret findings.

#### 2.8.2 Semistructured interviews

Research team members will analyze data from semistructured interviews at each time point separately using manifest content analysis.^52^ Manuscripts will be individually coded by two research team members not involved in GAB activities and subsequently categorized into subcategories and categories with a low degree of interpretation.^52^ Data analysis workshops will be held with wider research team members to facilitate peer examination. Trustworthiness^53^ will be established via disconfirming case analysis, peer examination, member checking, and audit trail, to help maintain reflexivity and minimize bias. To facilitate sense‐making and data interpretation, we will discuss findings with GAB members and coordinators.

### 2.9 Public contribution

This is a study protocol for involving guardians as public contributors; however, guardians did not contribute to developing and writing the study protocol.

### 2.10 Ethical considerations

We will follow the Declaration of Helsinki. Ethical approval has been obtained from Muhimbili National Hospital (MNH/IRB/VOL.I/2023/080), the Tanzania National Health Research Ethics Review Committee (NIMR/HQ/R.8A/Vol.IX/4544), and the Swedish Ethical Review Authority (2023–01381‐01). We will collect informed consent from all GAB members, who will be informed that they have the right to withdraw at any time without giving a reason. GAB members will be asked for separate consent for semistructured interviews, and coordinators will also provide consent. Data will be processed following the Swedish Patient Data Act (2008:355), the General Data Protection Regulation (EU 2016/679), and the Tanzanian National Institute of Medical Research (NIMR) research policy.

### 2.11 Reporting and dissemination

We will report GAB activities and results following the GRIPP2 checklist^39^ and the Standards for Reporting Qualitative Research (SRQR) checklist.^54^ Dissemination strategies will include plain language summaries, peer‐reviewed publications, and public exhibitions.

## 3 DISCUSSION

To the best of our knowledge, this protocol describes the first study designed to examine the acceptability, feasibility, and perceived impact of public contribution in healthcare research in Tanzania. We will set up an advisory board of guardians with lived experience, rather than local community leaders as common in LMIC settings.^37^ Public contribution activities will be led by Tanzanian research team members to better understand the local context and potential cultural sensitivities, develop trust, and utilize local knowledge from both guardians and the research team in Tanzania. Important contextual and cultural sensitivities may include religious beliefs,^55^ gender roles,^56^, ^57^ community stigma,^55^ urban and rural community resource divisions, lack of trust in government initiatives,^58^ and local customary processes including the impact of traditional community leaders.^59^ Careful consideration of these factors should facilitate the development of a research environment that utilizes robust scientific methods but remains open to diverse local perspectives, cultural traditions,^60^ and prioritizes guardians' needs and preferences.

Limitations include that guardians have not contributed to the study protocol design, limiting our ability to identify potential adaptations in advance to proposed public contribution activities that may have increased acceptability and feasibility. However, we will remain open to adapting public contribution activities in collaboration with GAB members. GAB members may have different views and opinions on discussed topics than each other and/or the research team. We will establish a scientific advisory group to review proposed research changes before making a final decision. This group will include the principal investigator (LvE), a GAB representative, and an academic expert not directly involved in the research. The ability of GAB members to influence decision‐making concerning certain research phases may be limited by factors such as funder requirements and scientific considerations. If GAB members make suggestions that cannot be implemented, they will be informed as to why.^61^ Public contribution in research stems from Western standards of scientific excellence. We aim to be flexible in how we conduct public contribution activities. However, the risk remains that Western‐centric norms may be imposed on the design and conduct of these activities. Despite these limitations, our planned public contribution activities and examination of the acceptability, feasibility, and perceived impact of these activities will help understand how to best embed public contribution in research in LMICs.

## AUTHOR CONTRIBUTIONS

**Faraja Chiwanga:** Methodology (supporting); project administration (equal); writing – original draft (equal); writing – review and editing (equal). **Joanne Woodford:** Methodology (equal); project administration (equal); visualization (equal); writing – original draft (equal); writing – review and editing (equal). **Golden Masika:** Visualization (supporting); writing – original draft (supporting). **David A. Richards:** Methodology (supporting); supervision (equal); writing – review and editing (supporting). **Victor Savi:** Writing – review and editing (supporting). **Louise von Essen:** Conceptualization (lead); funding acquisition (lead); methodology (lead); project administration (lead); writing – original draft (lead); writing – review and editing (lead).

## CONFLICT OF INTEREST STATEMENT

None.

## ETHICS STATEMENT

Ethical approval has been obtained from Muhimbili National Hospital (MNH/IRB/VOL.I/2023/080); the Tanzania National Health Research Ethics Review Committee (NIMR/HQ/R.8A/Vol.IX/4544); and the Swedish Ethical Review Authority (2023–01381‐01).

## Supporting information


Appendix S1.

## Data Availability

Data sharing is not applicable to this manuscript as no new data were created or analyzed.
